# Volumising thread lift technique for forehead augmentation

**DOI:** 10.1111/srt.13813

**Published:** 2024-06-14

**Authors:** Jovian Wan, Soo‐Bin Kim, Lisa Kwin Wah Chan, Kar Wai Alvin Lee, Hugues Cartier, Kyu‐Ho Yi

**Affiliations:** ^1^ Asia‐Pacific Aesthetic Academy Hong Kong Hong Kong; ^2^ Division in Anatomy and Developmental Biology Department of Oral Biology Human Identification Research Institute BK21 FOUR Project Yonsei University College of Dentistry Seodaemun‐gu Seoul South Korea; ^3^ EverKeen Medical Centre Hong Kong Hong Kong; ^4^ Centre Médical Saint Jean Arras France; ^5^ Maylin Clinic (Apgujeong) Seoul South Korea

Dear Editor

The forehead constitutes approximately one‐third of the face and significantly contributes to defining an individual's facial aesthetics. Men often have prominent supraorbital ridges and pronounced eyebrows, features traditionally associated with masculinity.^1^, ^2^ However, these characteristics are not exclusive to men and can also be present in women.^3^, ^4^ To address these features in women, some individuals choose to use filler injections above the supraorbital ridge to achieve a smoother facial contour.^5^, ^6^ However, caution is necessary when performing forehead procedures due to the presence of major blood vessels such as the supraorbital and supratrochlear arteries, which pose the risk of serious complications such as blindness and tissue necrosis.^6^, ^7^ Volumising thread lifts offer a safer alternative to mitigate these risks.^8^, ^9^ Thicker and firmer volume threads are commonly used for forehead augmentation, often employing techniques such as volumisation with N‐Scaffold (N‐Finders, Korea).

The aim of this article is to present a successful case of forehead augmentation utilising volumising thread lifts, and to demonstrate the thread lift insertion technique through a Video S1.

A 27‐year‐old female patient presented at the clinic, expressing concern about her forehead appearing “too flat”. Upon examination, it was observed that the patient's forehead lacked projection and had prominent supraorbital ridges. She had a history of receiving hyaluronic acid filler for chin augmentation 2 years ago but had never undergone any aesthetic treatments for her forehead. Additionally, she had no surgical history and medical history was unremarkable. The treating physician expressed concern regarding potential vascular complications due to age‐related fat atrophy. Subsequently, eight 18‐gauge 5 cm N‐Scaffold threads were inserted, with four on each side (see Video 1 and Figure 1). The patient underwent biweekly follow‐ups for 1 month following thread lift, during which no adverse events or complications were observed. A follow‐up photograph was obtained at the 1‐month mark to assess the outcomes (see Figure 2).

**FIGURE 1:**
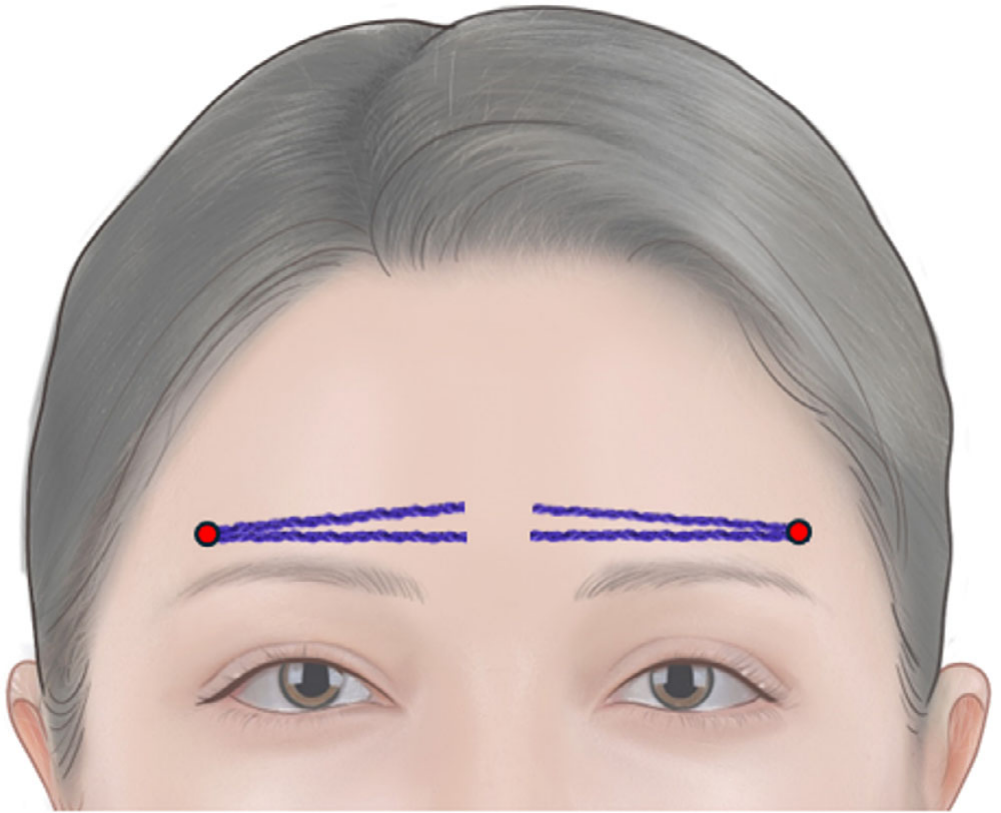
Schematic image of the insertion of N‐Scaffold for forehead augmentation. The entry point (marked with a red dot) was created 1 cm above the outer edge of the eyebrow, and the insertion was performed above the supraorbital ridge.

**FIGURE 2:**
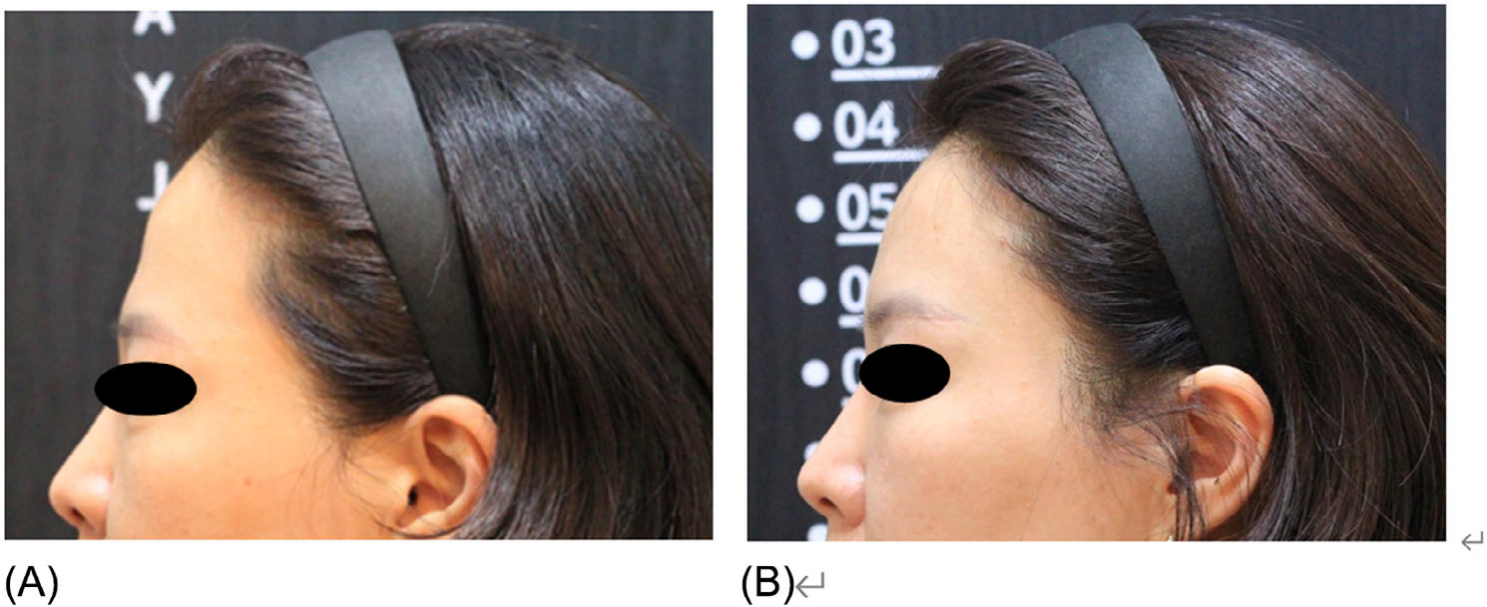
Forehead augmentation using volumising threads (N‐Scaffold, N‐finders, Korea) in a 27‐year‐old female. Digital photograph A display's the patient's appearance before the procedure, and photograph B is her appearance 1 month post‐treatment.

The patient expressed significant satisfaction with the outcomes, both immediately after the procedure and during the 1‐month follow‐up, noting a preference for the improved forehead projection and feeling more feminine.

Volume restoration in the forehead using filler injection has proven effective, although this region presents challenges due to the risk of serious complications.^5^, ^10^, ^11^, ^12^ Inexperienced injections frequently encounter challenges in achieving optimal results and refining injection techniques, which encompass identifying entry points and determining appropriate injection volumes.

In facial rejuvenation procedures, the primary focus is on achieving optimal results while minimising potential risks. The threads utilised in this case were absorbable polydioxanone (PDO) threads, characterised by a complex braided structure. Yi ^8^ explored the evolution of PDO volumising threads as a response to the evolving demands of facial rejuvenation procedures, primarily driven by age‐related changes in skin elasticity and volume. These braided threads, due to their layered composition, offer volume upon insertion without visible traces on the skin surface or palpable contours.^8^ PDO is commonly used in aesthetic medicine for facial rejuvenation and body contouring, with potential complications such as bruising, bleeding, and redness being typically minor and transient. Embedding PDO threads around fibrous connective tissue can enhance tissue thickness and reinforce soft tissue by connecting existing fibrous structures.^13^ Su et al.^13^ found that PDO may induce localised lipid metabolism, potentially promoting lipolysis. However, further research is warranted to fully understand this mechanism. Notably, in our case where the patient presented with age‐related fat atrophy in the forehead, these findings hold significance. While PDO threads are often used for minimally invasive lifting procedures and nasal reshaping, our technique introduces a novel application for forehead augmentation.^14^, ^15^, ^16^, ^17^, ^18^, ^19^So we introduce a new technique for using PDO thread lift for forehead augmentation.

Upon further examination of the complications linked to PDO threads in facial rejuvenation procedures, Bertossi et al.^20^ and Niu et al.^21^ have highlighted various adverse events, including displacement of barbed sutures, erythema, infection, skin dimpling, and facial stiffness. Older patients are more susceptible to certain complications, such as dimpling and infection.^21^ Moreover, severe complications like open wounds, abscesses, and skin necrosis have been documented in some cases.^22^ Inflammatory reactions and bacterial complications, such as facial abscesses, underscore the criticality of employing proper technique and medical supervision during PDO thread lift procedures.^23^ Additionally, the possibility of delayed absorption or non‐absorption of PDO threads may result in prolonged inflammation or other adverse effects, emphasising the importance of diligent monitoring and management.^24^

In conclusion, our case report demonstrates the efficacy of forehead augmentation using volumising PDO thread lift as a safe and effective procedure in facial rejuvenation. The patient's satisfaction with the results underscores the success of this intervention in achieving desired aesthetic outcomes. Despite potential complications, the benefits of PDO thread lifts in forehead augmentation offer a promising avenue for minimally invasive facial rejuvenation procedures. Further research is warranted to evaluate long‐term safety and efficacy in PDO thread lift procedures for facial rejuvenation.

## CONFLICT OF INTEREST STATEMENT

The authors declared no potential conflicts of interest with respect to the research, authorship, and publication of this article. This study was conducted in compliance with the principles set forth in the Declaration of Helsinki.

## Supporting information

Supporting information

## Data Availability

The data that support the findings of this study are available from the corresponding author upon reasonable request.
